# Prizes Awarded by the Academy of Medicine of Paris, for 1859

**Published:** 1860-05

**Authors:** 


PRIZES AWARDED BY THE ACADEMY OF MEDICINE OF PARIS FOR THE YEAR 1859.—The Montyon prize for experimental physiology was awarded to M. Pasteur, for his researches on fermentation, and a certificate to M. Ollier for his work upon transplantation of the periosteum. The committee has postponed to next year the examination of two other works on physiology: one by M. Budge on the nervous system, the other by M. Corvisart on digestion.

The Montyon prize for medicine and surgery has not been awarded, but certificates of the value of three hundred dollars have been granted—1st. To M. Béhier, for his book on "Puerperal Fever." 2d. To M. Gallois, for his memoir on "Oxalate of Lime in the Urine, Gravel, and Calculi." 3d. To M. Giraud-Teulon, for his work "On the Principles of Animal Mechanism, or the Study of Locomotion in Man and Vertebrate Animals." 4th. To M. Luschka, for his monograph "On Hemidiathroses of the Human Body." 5th. To M. Legendre, for his essay "On Some Rare Varieties of Femoral Hernia." 6th. To M. Marcé, for his book "On the Insanity of Pregnant Women, those in the Puerperal State, or Nursing." The committee also made honorable mention of several other works.

The Bréant prize for the best mode of curing cholera had fourteen competitors, but was not awarded. The Yecker prize for organic chemistry was granted—1st. To M. Wurtz, for his work "On Glycol and its Derivatives, and recently discovered Oxygenated Alkalies." 2d. To M. Cahours, for his work "On Organic Radicals." The value of the first was seven hundred dollars, of the last five hundred dollars.

